# Mechanisms of rosuvastatin-related acute kidney injury following cardiac surgery: the STICS trial

**DOI:** 10.1093/eurheartj/ehad640

**Published:** 2023-10-04

**Authors:** Rohan S Wijesurendra, Rebecca Sardell, Raja Jayaram, Nathan Samuel, Zhengming Chen, Natalie Staplin, Rory Collins, Zhe Zheng, Richard Haynes, Michael Hill, Jonathan Emberson, Barbara Casadei

**Affiliations:** Division of Cardiovascular Medicine, BHF Centre of Research Excellence, University of Oxford, UK; Clinical Trial Service Unit and Epidemiological Studies Unit, Nuffield Department of Population Health, University of Oxford, Oxford, UK; Clinical Trial Service Unit and Epidemiological Studies Unit, Nuffield Department of Population Health, University of Oxford, Oxford, UK; Division of Cardiovascular Medicine, BHF Centre of Research Excellence, University of Oxford, UK; Clinical Trial Service Unit and Epidemiological Studies Unit, Nuffield Department of Population Health, University of Oxford, Oxford, UK; Clinical Trial Service Unit and Epidemiological Studies Unit, Nuffield Department of Population Health, University of Oxford, Oxford, UK; Clinical Trial Service Unit and Epidemiological Studies Unit, Nuffield Department of Population Health, University of Oxford, Oxford, UK; Medical Research Council Population Health Research Unit, Nuffield Department of Population Health, University of Oxford, Oxford, UK; Clinical Trial Service Unit and Epidemiological Studies Unit, Nuffield Department of Population Health, University of Oxford, Oxford, UK; Department of Cardiovascular Surgery, State Key Laboratory of Cardiovascular Disease, National Clinical Research Center of Cardiovascular Diseases, Fuwai Hospital, National Center for Cardiovascular Diseases, Chinese Academy of Medical Sciences and Peking Union Medical College, Beijing, China; Clinical Trial Service Unit and Epidemiological Studies Unit, Nuffield Department of Population Health, University of Oxford, Oxford, UK; Medical Research Council Population Health Research Unit, Nuffield Department of Population Health, University of Oxford, Oxford, UK; Clinical Trial Service Unit and Epidemiological Studies Unit, Nuffield Department of Population Health, University of Oxford, Oxford, UK; Medical Research Council Population Health Research Unit, Nuffield Department of Population Health, University of Oxford, Oxford, UK; Clinical Trial Service Unit and Epidemiological Studies Unit, Nuffield Department of Population Health, University of Oxford, Oxford, UK; Medical Research Council Population Health Research Unit, Nuffield Department of Population Health, University of Oxford, Oxford, UK; Division of Cardiovascular Medicine, BHF Centre of Research Excellence, University of Oxford, UK

**Keywords:** Statin, Acute kidney injury, Cardiac surgery, KIM-1

## Introduction

The Statin Therapy In Cardiac Surgery (STICS) trial was a randomized, double-blind, placebo-controlled trial investigating the effects of perioperative rosuvastatin on postoperative atrial fibrillation and cardiac injury in patients undergoing cardiac surgery.^1^ While rosuvastatin did not significantly affect either outcome, acute kidney injury (AKI) was unexpectedly and significantly more common in rosuvastatin-allocated patients.^1^

A smaller trial of high-dose perioperative atorvastatin in patients undergoing cardiac surgery also did not support initiation of statin therapy to prevent postoperative AKI and was stopped prematurely with a null result after 615 patients had been randomized.^2^ Other similar trials have reported variable results, but these were all small (≤200 patients each), and many had additional limitations.^3^

To understand the mechanism of postoperative AKI, we undertook further analysis of the STICS samples, including measurement of cystatin C, which may have advantages over creatinine for diagnosis of AKI in this context,^4^ and several other biomarkers relevant to inflammation and AKI.

## Methods

The methodology and primary results of STICS have been published previously^1^; briefly, 1922 patients undergoing elective cardiac surgery were randomized to rosuvastatin 20 mg once daily or placebo for ≤8 days before surgery and 5 days thereafter.

Creatinine, cystatin C, growth differentiation factor 15 (GDF-15), interleukin-6 (IL-6), procalcitonin (PCT), placental growth factor (PLGF), kidney injury molecule-1 (KIM-1), and neutrophil gelatinase–associated lipocalin (NGAL) were measured at baseline and after surgery (at an interval of 48 h for creatinine, cystatin C, KIM-1, and NGAL and 6 h for GDF-15, IL-6, PLGF, and PCT).

Acute kidney injury by serum creatinine was defined and classified as Stages 1–3 based on KDIGO criteria^5^ (without data on urine output). Acute kidney injury was separately defined using serum cystatin C,^6^ using the same fold increases from baseline as for creatinine. A sensitivity analysis included an alternative definition of cystatin C–defined Stage 1 AKI (increase from baseline by a factor of at least 1.1 to <2).

Randomized comparisons were performed according to the intention-to-treat principle. Odds ratios (OR) and 95% confidence intervals (CI) were used for between-group comparisons of postoperative AKI. Analysis of covariance was used to compare biomarkers after surgery, with adjustment for baseline values. Analyses were performed on the log scale for all biomarkers and then transformed back to the original scale as geometric means. For dichotomous outcomes, patients with missing data were assumed not to have had the outcome. Missing values for biomarkers were estimated by means of multiple imputations, with 10 replicate sets and combination across sets with the use of Rubin’s methods.^7^

Full details of methods and assay performance have been reported separately.^8^

## Results

Baseline characteristics of the trial population have been published previously.^1^ Estimated glomerular filtration rate (eGFR) was 90 ± 15 mL/min/1.73 m^2^. Overall, 66% of patients were statin naïve, 31% had diabetes, 4.5% had eGFR < 60 mL/min/1.73 m^2^, and 40% were receiving an angiotensin-converting enzyme inhibitor or angiotensin receptor blocker. Over 91% of baseline and follow-up biomarker results were available.^8^

**Figure 1:**
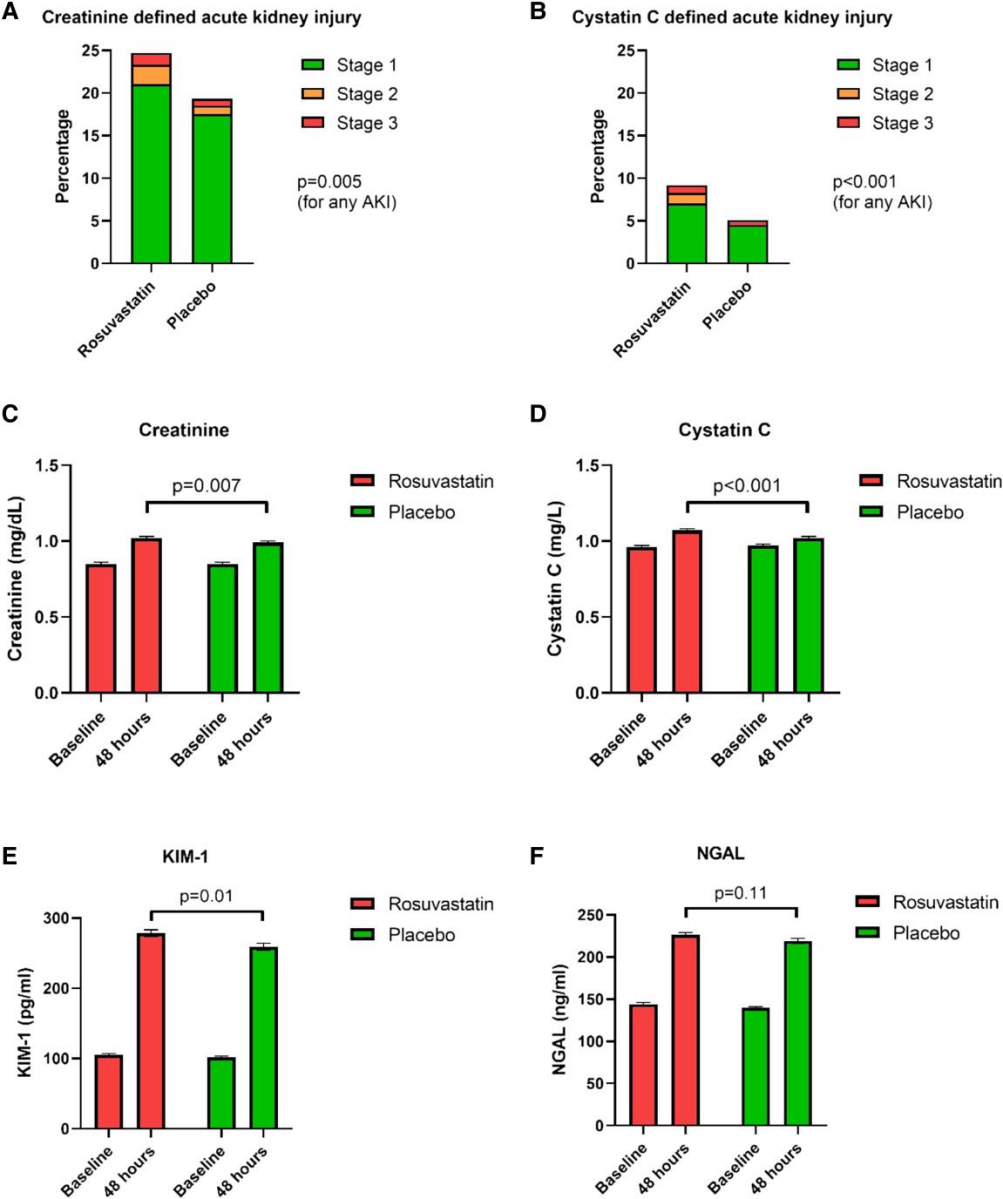
Effect of allocation to rosuvastatin on creatinine-defined and cystatin C–defined acute kidney injury and on postoperative creatinine and cystatin C levels. The upper panel shows bar graphs indicating proportion of acute kidney injury Stages 1–3 at 48 h postoperatively in the rosuvastatin and placebo groups, defined by creatinine (*A*, as reported in Ref. 1) and cystatin C (*B*). Participants missing cystatin C or creatinine were assumed not to have acute kidney injury unless they had undergone renal replacement therapy. The lower panel shows bar graphs indicating levels of creatinine (*C*), cystatin C (*D*), kidney injury molecule-1 (*E*), and neutrophil gelatinase–associated lipocalin (*F*) at baseline and at 48 h postoperatively in the rosuvastatin and placebo groups. Bars show geometric means with approximate ± SE. *P*-values were derived from analysis of covariance with adjustment for the baseline value with any missing data imputed with the use of multiple imputations.

The incidence of creatinine-defined AKI was greater in the rosuvastatin group than in the placebo group (24.7% vs. 19.3%, OR 1.37, 95% CI 1.10–1.70, *P* = .005; *Figure 1A*)^1^, as was the incidence of cystatin C–defined AKI (9.2% vs. 5.1%, OR 1.86, 95% CI 1.29–2.67, *P* < .001; *Figure 1B*). When the cystatin C definition of AKI was expanded to include an increase from baseline in the cystatin C level by a factor of at least 1.1, the incidence of AKI was 46.0% vs. 36.7% (OR 1.47, 95% CI 1.23–1.77, *P* < .001). Creatinine and cystatin C levels in both treatment groups increased from baseline to 48 h after surgery; however, baseline-adjusted creatinine and cystatin C levels at 48 h were significantly higher in rosuvastatin-allocated than placebo-allocated patients (1.02 ± 0.01 mg/dL vs. 0.99 ± 0.01 mg/dL, *P* = .007, *Figure 1C*, and 1.07 ± 0.01 mg/L vs. 1.02 ± 0.01 mg/L, *P* < .001, *Figure 1D*, respectively). The breakdown of absolute excess in cystatin C–defined AKI in the rosuvastatin group compared to the placebo group was the following: stage 1 (2.5 ± 1.1%); stage 2 (1.3 ± 0.4%); and stage 3 (0.3 ± 0.4%).

Concentrations of GDF-15, IL-6, PCT, PLGF, KIM-1, and NGAL were all substantially higher after surgery than at baseline (all *P* < .001). For KIM-1, the rise was significantly higher in patients allocated to rosuvastatin compared to placebo (baseline-adjusted mean KIM-1 concentration at 48 h: 278 ± 5 pg/mL vs. 259 ± 5 pg/mL, *P* = .01; *Figure 1E*), whereas there was no significant difference between the groups in the rise for GDF-15, IL-6, PCT, PLGF, and NGAL (*Figure 1F*).

## Discussion

Allocation to rosuvastatin compared to placebo increased the absolute risk of postoperative AKI, however defined, by 4%–5% in patients undergoing cardiac surgery. The postoperative concentration of KIM-1 was also higher in patients allocated to rosuvastatin compared to placebo, suggesting that allocation to rosuvastatin may exacerbate renal proximal tubular injury^9^ in the context of cardiac surgery. In contrast, there were no significant differences in NGAL, GDF-15, IL-6, PCT, or PLGF between the groups, suggesting that the adverse effect of perioperative rosuvastatin on renal function may be independent of systemic inflammation and renal epithelial tissue injury.^10^ Although rosuvastatin also led to a significant increase in postoperative creatine kinase, adjustment for this did not materially change the estimated effect of allocation to rosuvastatin on postoperative AKI, which remained significant in fully adjusted multivariable analyses.^8^

The finding that perioperative rosuvastatin increases the risk of AKI after cardiac surgery is highly clinically relevant, particularly in the context of a null effect on postoperative complications.^1^ A meta-analysis of 11 trials of perioperative statin therapy in cardiac surgery (including STICS) confirmed a higher incidence of AKI in cardiac surgery patients receiving perioperative statins compared to control.^3^ STICS contributed the strongest evidence on this topic (accounting for 423 of 650 AKI events) and provided a unique opportunity to further investigate the mechanisms of AKI in the present study.

Although the majority of the excess AKI following rosuvastatin use in STICS was relatively minor (Stage 1), incompletely resolved AKI could lead to chronic kidney disease and increased risk of future cardiovascular events. In the absence of further trial data, temporary perioperative statin cessation in patients undergoing cardiac surgery may be a reasonable option to consider on a case-by-case basis.

## Data Availability

The data underlying this article will be shared upon reasonable request to the corresponding author.

## Pre-registered Clinical Trial Number

NCT01573143.
